# Ventral-approach augmented nontransected anastomotic (vANTA) urethroplasty for bulbar urethral strictures: a single-center experience

**DOI:** 10.55730/1300-0144.5848

**Published:** 2024-05-11

**Authors:** Musab Ali KUTLUHAN, Sait AYGÜN, Selman ÜNAL, Asım ÖZAYAR, Emrah OKULU, Kemal ENER, Önder KAYIGİL

**Affiliations:** 1 Department of Urology, Faculty of Medicine, Ankara Yıldırım Beyazıt University, Ankara, Turkiye; 2 Department of Urology, Ürgüp State Hospital, Nevşehir, Turkiye

**Keywords:** Urethral stricture, urethroplasty, sexual function, quality of life, urinary function

## Abstract

**Background/aim:**

This study describes ventral-approach augmented nontransected anastomotic (vANTA) urethroplasty and presents the preliminary functional results of patients treated with this technique.

**Materials and methods:**

Twenty-three patients who underwent vANTA urethroplasty were included in the study. Stricture location, stricture length, preoperative uroflowmetry parameters (maximum flow rate (Qmax) and mean flow rate (Qmean)), preoperative International Index of Erectile Function (IIEF)-5 scores, operation time, postoperative complications, length of hospital stay, and follow-up periods were recorded. The Qmax, Qmean, and IIEF-5 scores of the patients were recorded again in the second and twelfth postoperative months. Preoperative and postoperative Qmax values and IIEF-5 scores were compared. Kaplan–Meier survival analysis was performed to demonstrate recurrence-free survival.

**Results:**

The mean age of the patients included in the study was 52.1 ± 16.9 years. Mean stricture length was 2.5 ± 0.5 cm. There was a statistically significant difference between preoperative and 2-month postoperative uroflowmetry Qmax values (6.9 (0.0–14.5) vs. 18.5 (5.5–41.5) mL/s; p < 0.001). There was no statistically significant difference in preoperative and 2-month postoperative IIEF-5 scores (p > 0.05). There was a statistically significant difference between preoperative and 1-year postoperative median Qmax values (7.2 (0.0–12.3) vs. 17.4 (11.2–24.3) mL/s; p = 0.001). There was no statistically significant difference between preoperative and 1-year postoperative IIEF-5 scores (p > 0.05). According to Kaplan–Meier recurrence-free survival analysis, the recurrence-free survival rate at 6 months was 95.7%

**Conclusion:**

In cases of bulbar urethral strictures, vANTA urethroplasty is an effective treatment option with limited postoperative complications. Preserving the underlying corpus spongiosum is important to avoid impaired sexual function.

## 1. Introduction

The incidence of urethral strictures has increased with the increasing use of retrograde endoscopic interventions [1]. Urethral strictures are mostly seen in the bulbar urethra and endoscopic interventions may fail [2]. Urethroplasty has high success rates in cases of bulbar urethral strictures [3]. Various augmentation techniques with buccal mucosa grafts (BMGs) have been used in the treatment of bulbar urethral strictures, including dorsal onlay, dorsolateral onlay, and other augmentation approaches [4–7].

Together with developments in augmentation techniques, revisions have also been made to anastomotic urethroplasty techniques. Because of concerns about sexual outcomes after transecting urethroplasty, Andrich and Mundy performed nontransecting anastomotic urethroplasty [8] in cases of short bulbar urethral strictures. Welk and Kodama subsequently described augmented nontransected anastomotic urethroplasty (ANTA) [9]. This technique is applied in cases of severe obliterative urethral strictures. With this technique, dorsal urethrotomy is performed and the obliterated urethral mucosa is excised. Healthy urethral mucosa margins are anastomosed end-to-end and a BMG is placed with dorsal onlay. In our clinical practice, we frequently use the ventral approach for bulbar urethral strictures. ANTA can be applied via a ventral approach in cases of severe obliterative strictures or mucosal irregularities. In our technique, we apply dorsal urethral mucosal excision and end-to-end mucosal anastomosis through a ventral approach and place a ventral BMG in patients with bulbar urethral strictures. This technique is a modified version of the ANTA.

The aim of the present study is to describe ventral-approach augmented nontransected anastomotic (vANTA) urethroplasty and present our preliminary results.

## 2. Materials and methods

### 2.1. Patient selection and study design

After local ethics committee approval was obtained (#26379996/22), patients undergoing perineal open urethroplasty due to a bulbar urethral stricture were screened. Twenty-three patients who underwent vANTA urethroplasty between May 2021 and August 2023 were included in the study. Age, comorbidities, etiological factors of the urethral stricture, and previous procedures were recorded for all patients. In addition, stricture location, stricture length, preoperative uroflowmetry parameters (maximum flow rate (Qmax) and mean flow rate (Qmean)), preoperative International Index of Erectile Function (IIEF)-5 scores, operation time, postoperative complications, length of hospital stay, and follow-up information were recorded. The Qmax, Qmean, and IIEF-5 scores of the patients were recorded again in the second and twelfth postoperative months and data on complications were collected during follow-up visits in the sixth month.

### 2.2. Inclusion and exclusion criteria

Patients who underwent vANTA urethroplasty due to bulbar urethral stricture and had postoperative follow-up of at least 6 months were included in the study. Patients who had missing data in their charts were excluded.

### 2.3. Surgical technique

**Figure 1:**
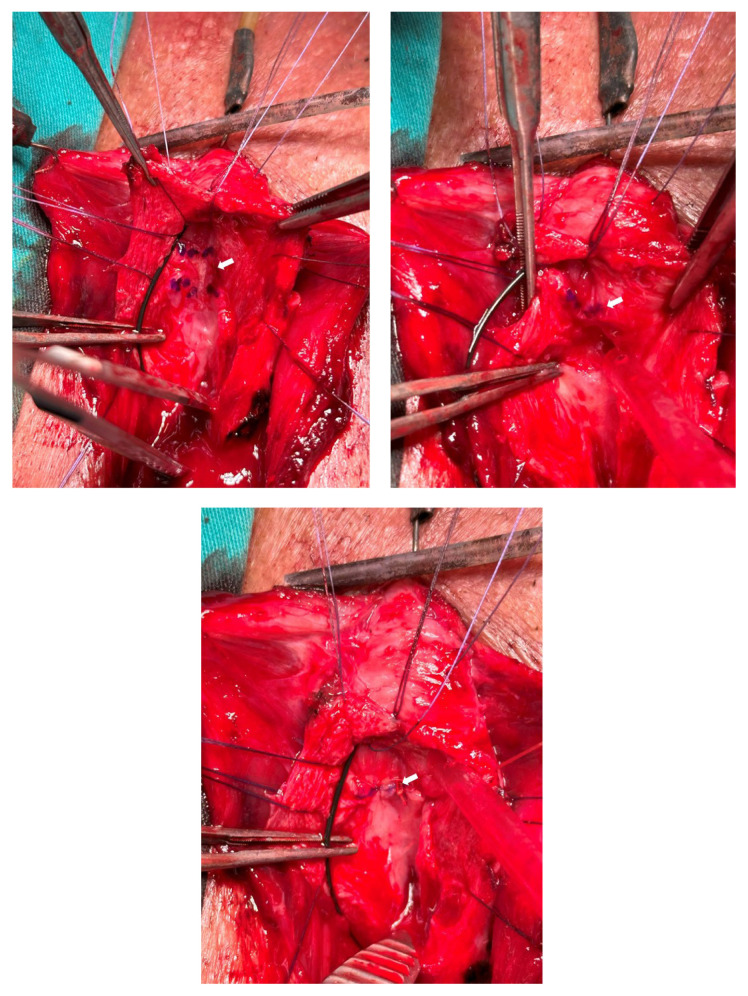
a) Very tight section of the stricture is marked; b) mucosa is excised, preserving the underlying spongiosum; c) mucosa-to-mucosa anastomosis with 5/0 rapid Vicryl. White arrows show the tightest urethral segment.

**Figure 2:**
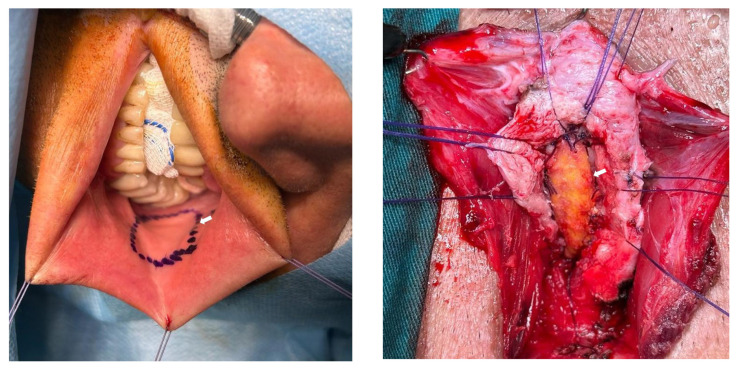
a) The Stenon canal and buccal mucosal graft incision line are marked; b) placement of the ventral-onlay buccal mucosa graft. White arrows show the buccal mucosa graft that was harvested and placed on the urethra.

All surgical procedures were performed by a single reconstructive urethral surgeon (M.A.K.). After endoscopic evaluation with a semirigid 7-F ureterorenoscope, a guidewire catheter was placed in the bladder and methylene blue was injected through the urethra. A vertical perineal incision was subsequently made. The bulbospongiosus muscle was visualized and cut from the midline. The bulbar urethra was revealed. After identifying the stricture site with a 16-F Nelaton catheter, the urethra was opened ventrally vertically (ventral urethrotomy). The mucosa of the tightest urethral segment was marked, as shown in Figure 1a, and it was excised while preserving the underlying corpus spongiosum (Figure 1b). Proximal and distal urethral mucosa ends were anastomosed dorsally end-to-end (nontransecting anastomosis) (Figure 1c). For dorsal mucosa–mucosa anastomosis, 5/0 rapid Vicryl was used. For the BMG harvest, three hanging sutures were placed on the edges of the lips. The Stenon canal and the BMG incision line were marked with a marker pen (Figure 2a). An adrenaline solution (1:100,000) was injected beneath the mucosa to ease the dissection. The required BMG was obtained according to the length of the stricture. The distal and proximal ends of the BMG were anastomosed ventrally to the distal and proximal ends of the urethral mucosa using 4/0 Vicryl sutures. After the 16-F silicone catheter was placed in the bladder, the lateral openings were closed using 4/0 Vicryl for BMG–urethral mucosa anastomosis (ventral augmentation) (Figure 2b).

### 2.4. Follow-up procedure

**Figure 3:**
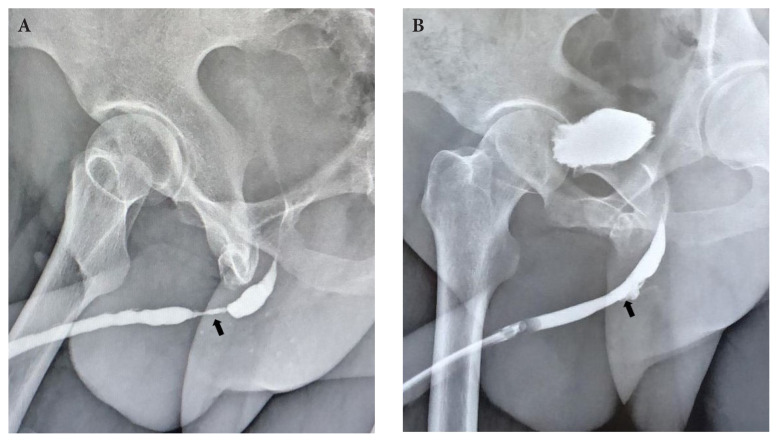
a) Preoperative retrograde urethrography; b) postoperative retrograde urethrography in week 3 of follow-up. Black arrows show the location of the urethral stricture.

The data of all patients were recorded prospectively from the first case. Patients were catheterized for 3 weeks. All patients underwent preoperative (Figure 3a) and 3-week postoperative retrograde urethrography (RUG) (Figure 3b). In the event of urinary extravasation, catheter duration was extended. Postoperative voiding and erectile function evaluations were performed routinely at the end of the second postoperative month. Subsequently, 6- and 12-month postoperative evaluations were performed. Retrograde endoscopic evaluation was performed for all patients who described voiding dysfunction after postoperative catheter removal.

### 2.5. Definition of success

Success was defined as the absence of any voiding symptoms without the need for any postoperative procedures (i.e., direct vision internal urethrotomy or urethral dilatation).

### 2.6. Statistical analyses

Statistical analyses were performed using IBM SPSS Statistics 24.0. Continuous variables were expressed as mean, standard deviation, minimum, and maximum. The groups were compared with the Wilcoxon test because the data were not normally distributed. Categorical variables were expressed as frequencies and percentages. Kaplan–Meier survival analysis was performed to demonstrate recurrence-free survival. Statistical significance was accepted at p < 0.05.

## 3. Results

The mean age of the patients in this study was 52.1 ± 16.9 years. Mean stricture length was 2.5 ± 0.5 cm. The most common stricture location was the middle bulbar level (78.3%). The clinical characteristics of the patients are summarized in Table 1.

There was a significant difference between preoperative and 2-month postoperative median Qmax values (6.9 (0.0–14.5) vs. 18.5 (5.5–41.5) mL/s, respectively; p < 0.001). In long-term evaluations, there was a significant difference between preoperative and 1-year postoperative median Qmax values (7.2 (0.0–12.3) vs. 17.4 (11.2–24.3) mL/s, respectively; p = 0.001).

There was no significant effect of vANTA urethroplasty on either short- or long-term erectile functions as evaluated by the IIEF-5 (p > 0.05 for the comparison of preoperative results with those of 2-month and 1-year follow-up). No patient without preoperative erectile dysfunction (ED) developed early postoperative ED. Voiding and erectile functions during follow-up are summarized in Table 2.

Urethral stricture recurrence occurred in 2 of 23 cases as observed in the sixth and eighth postoperative months. The recurrences developed as fibrotic rings at the proximal or distal end of the graft. Direct vision internal urethrotomy was performed successfully for these patients. According to Kaplan–Meier recurrence-free survival analysis, 6-month recurrence-free survival was 95.7%.

Postoperative surgical complications were observed in three cases as follows: urinary tract infection in one patient, wound infection and dehiscence in one patient, and urinary extravasation in one patient. The urinary tract infection was managed with appropriate antibiotic therapy after urine culture. Wound dehiscence was managed with secondary closure after antibiotic therapy. Urethral catheter duration was extended for 1 week for one patient who had urinary extravasation by RUG in the third postoperative week.

## 4. Discussion

Nontransecting anastomosis techniques have evolved over the years and are now being used to treat bulbar urethral strictures [10]. In cases of bulbar urethral strictures, we usually prefer a ventral approach in our practice. If the stricture length is more than 2 cm, we usually prefer ventral-onlay BMG urethroplasty. In some cases, after a ventral midline urethrotomy, we encounter severe mucosal lumen narrowing, mucosal scars, or mucosal patch areas in some sites of the stricture. If the very tight obliterative stricture segment is not longer than 2 cm, we perform vANTA urethroplasty. Our preliminary results are promising. The short-term recurrence-free rate was 95.7 in this study. In addition, none of our patients had adverse sexual complications or postoperative ED, which is an important finding, especially for younger and/or sexually active patients.

Nontransecting dorsal mucosal anastomosis plus ventral-onlay graft urethroplasty was described by Palminteri et al. [11]. They preferred this technique for tight bulbar strictures as we do. In that study, the median follow-up period was 58 months and the mean stricture length was 1 cm. The success rate was 82%, which is promising. In addition, none of the patients had postoperative sexual impairment [11]. Recently, Marks et al. described a similar technique referred to as mucomucosal anastomotic nontransecting augmentation (MANTA) urethroplasty [12]. According to their results, the success rate was 93% with median follow-up of 41 months [12]. In our study, the mean follow-up duration was 11.6 ± 5.1 (6.0–23.0) months and the mean stricture length was 2.5 ± 0.5 (2.0–3.5) cm. Our success rate was 91%, which is compatible with the literature. According to Kaplan–Meier survival analyses, the 6-month recurrence-free survival rate was 95.7%. No patients without preoperative ED developed postoperative ED during follow-up.

There are some advantages and disadvantages of ventral-onlay BMG urethroplasty in treating bulbar urethral strictures. The major advantage of this approach is that it is technically easier than the dorsal or dorsolateral approach. Furthermore, complete mobilization of the urethra, which can potentially impair the neurovascular supply of the urethra, is unnecessary. In addition, ventral urethrotomy allows wide-angle evaluations of the urethral mucosa to determine the tightest section. On the other hand, some disadvantages exist regarding ventral-onlay BMG urethroplasty. The major disadvantage of this approach is the potential for a large amount of blood loss due to the excision of the corpus spongiosum, which is thicker in the bulbar urethra. However, stay sutures in the urethral plate are efficient in controlling bleeding and none of our patients needed a blood transfusion. Second, this approach should not be performed for patients with severe fibrosis (traumatic strictures, radiotherapy-related strictures, etc.). Third, there is a risk of diverticulum formation. It is important to optimize the BMG width to prevent urethral diverticulum. Finally, this approach should not be applied for penile urethral strictures because the corpus spongiosum is thinner around the penile urethra.

According to the literature, ventral-onlay BMG urethroplasty has excellent results in the treatment of bulbar urethral strictures. Barbagli et al. stated that ventral-onlay BMG urethroplasty for bulbar urethral strictures had an 85% success rate [13]. They also noted that it was not necessary to excise tight segments of urethral mucosa in a nontransecting fashion. Instead, they sutured the BMG directly to the spongiosum tissue in the narrowest part of the stricture. They concluded that there was no significant difference in success rates between wide and narrow urethral plates. In our opinion, excision of the tight segment can also be performed for bulbar urethral strictures because there could be regeneration of the urethral mucosa to restore the urethral plate. However, in some cases, there is no chance of anastomosing the BMG with the urethral mucosa even with a one-sided application. Thus, we usually excise the tightest segment of the stricture in a nontransecting fashion. We obtain an intact dorsal urethral mucosal surface using dorsal mucosa–mucosa anastomosis.

It was previously demonstrated that the nontransecting approach had better sexual outcomes than the transecting technique with similar recurrence rates [14]. Palminteri et al. evaluated the impact of ventral BMG urethroplasty on sexual function. They concluded that minimally invasive ventral graft urethroplasty does not cause sexual complications in short-term follow-up apart from postejaculation dribbling [15]. In a comprehensive review, it was stated that in bulbar reconstructions, graft augmentation techniques seem to have fewer effects on sexual functions than excision and primary anastomotic techniques [16]. One randomized controlled study demonstrated that in terms of erectile function as measured by the IIEF-5 score, there were no significant differences between transecting anastomosis and BMG urethroplasty, but more penile complications (shortening, reduced glans filling) were encountered after transecting anastomosis, which is important, especially for younger and sexually active patients [17]. In our preliminary series, most of our patients were sexually active and none of them had ED after surgery.

Our study has some limitations. First, this was a retrospective chart review study, which entails potential selection bias, confounding variables, and potential inaccuracies in medical records. However, in our center, the data of all patients who underwent urethroplasty were recorded prospectively from the first case. Second, we did not compare vANTA outcomes with ventral-onlay BMG urethroplasty because our aim in this study was to describe the technique and present our preliminary results. We will compare the two techniques as the number of patients in both arms increases. Third, we only evaluated erectile function, not ejaculatory function or other sexual functions.

In conclusion, according to our results, vANTA urethroplasty is an effective option in the treatment of bulbar urethral strictures, offering the benefit of limited postoperative complications. Preserving the underlying corpus spongiosum is important to avoid erectile function impairment. Prospective randomized controlled studies with large sample sizes are needed to reach a firm conclusion.

## Figures and Tables

**Table 1:** Characteristics of the patients.

Variables	
Number of patients	23
Age, years (range)	52.1 ± 16.9 (22.0–88.0)
Etiology, n (%)	23 (100.0%)
Catheterization	4 (17.4%)
TUR-P	12 (52.1%)
URS	2 (8.7%)
Infection	4 (17.4%)
Idiopathic	1 (4.3%)
Previous intervention history, n (%)	
Open urethroplasty	0
VIU	23 (100.0%)
Dilatation	4 (17.3%)
None	0
Mean stricture length, cm (range)	2.5 ± 0.5 (2.0–3.5)
Stricture location, n (%)	23 (100.0%)
Distal bulbar	3 (13.0%)
Middle bulbar	18 (78.3%)
Proximal bulbar	2 (8.7%)
Mean duration of surgery, h (range)	2.1 ± 0.5 (1.5–3.0)
Mean follow-up time, months (range)	11.6 ± 5.1 (6.0–23.0)
Success rate, n (%)	21 (91.3%)

DM: Diabetes mellitus; CVD: cardiovascular disease; TUR-P: transurethral resection of the prostate; URS: ureterorenoscopy; VIU: visual internal urethrotomy.

**Table 2:** Comparisons of preoperative and postoperative functional parameters.

Variables	n = 23	p
Preoperative UFM Qmax (mL/s), median (range)	6.9 (0.0–14.5)	<0.001
Postoperative 2nd-month UFM Qmax (mL/s), median (range)	18.5 (5.5–41.5)	
Preoperative IIEF-5 score, median (range)	21.0 (4.0–29.0)	0.116
Postoperative 2nd-month IIEF-5 score, median (range)	19.5 (4.0–28.0)	
	n = 12	p
Preoperative UFM Qmax (mL/s), median (range)	7.2 (0.0–12.3)	0.001
Postoperative 1st-year UFM Qmax (mL/s), median (range)	17.4 (11.2–24.3)	
Preoperative IIEF-5 score, median (range)	20.0 (4.0–29.0)	0.317
Postoperative 1st-year IIEF-5 score, median (range)	19.5 (4.0–29.0)	

UFM: Uroflowmetry; Qmax: maximum flow rate; IIEF: International Index of Erectile Function.
